# Supplementary material for: Species-specific enamel differences in hardness and abrasion resistance between the permanent incisors of cattle (Bos primigenius taurus) and the ever-growing incisors of nutria (Myocastor coypus)
Source: PLoS One. 2022 Mar 17;17(3):e0265237. doi: 10.1371/journal.pone.0265237 (PMC8929658; doi:10.1371/journal.pone.0265237)
Supplement: Attachment — Submitted filename: Clauss_ReplyLetter_211222.pdf [file pone.0265237.s002.pdf]

PONE-D-21-35327
Reply to Reviewers

Dear Cyril Charles,

thank you for giving us the opportunity to revise our manuscript and respond to the Reviewers' comments.

Please find attached the revised manuscript with all revised text in grey shading. Our response to specific reviewer comments are given at the bottom of this letter.

We thank you for your time and hope the manuscript now meets the requirements of PLoS One.

Sincerely,

Marcus Clauss

See detailed responses to reviewers down below

# Reviewer 1:

Reviewer #1: This very interesting paper deals with the influence of enamel hardness on dental wear comparing two mammal species, cattle and nutria, presenting different dental characteristics. This study focusing on incisors provides counter-intuitive but intriguing results showing that dental wear tends to be more species-dependent than hardness-dependent and that other factors must be taken into account, which is discussed accordingly.
I have also a few minor comments on the manuscript, which deserves to be published pending these modifications.

| Reviewer's comment (lines refer to the original submission) | Our response | Changes made in the text (lines refer to the revised version) |
|---|---|---|
| As a result, this work raises a lot of questions on the differences observed between the two species, and some of them could probably be answered by studying closer taxa having more similar incisor properties (except hardness, and then crown height). My main (but sole) concern is thus the choice of species and of the tooth locus, which should be more accurately explained in relation to the main question of the study based on brachyodont vs hypselodont taxa. I understand that the availability of large samples of cattle and nutria from slaughterhouses could be one of the main criteria for having chosen these two species. However, they present too many different incisor characteristics (some of them are mentioned in the paper) in addition to crown height (e.g. only lower incisors in cattle, only one pair of incisors with only labial enamel in nutria, different enamel thicknesses and microstructures, high iron oxide enrichment in the enamel of nutria, different bite forces and jaw motions) which likely have an impact on wear and render these results more difficult to compare from a biomechanical viewpoint. For these reasons, study of molars (presenting less differences) or comparisons of cattle and nutria with other bovids (or cervids), rodents or with equids (having hypsodont incisors) could have been more appropriate to reduce the potential effect of other factors on wear compared to hardness or crown height. | This is a good point and we included this in the discussion. | addition made l. 305: For the present experiment, we deliberately chose two species for which dental material was relatively easily available (from slaughterhouses), and in which we expected a very distinct difference in hardness due to the non-ever-growing / hypselodont dichotomy outlined in the introduction. For more detailed investigations on the interplay of enamel hardness and abrasion, several considerations – apart from simply increasing the number of investigated species – apply. For example, it might be more interesting to investigate phylogenetically more closely related species, excluding large differences in terms of oral anatomy and chewing physiology, yet still putatively differing in enamel hardness, for example bovids and giraffids that appear particularly vulnerable to dental wear [49]. Additionally, expanding the method to enamel from cheek teeth may be more relevant for non-gnawing mammalian taxa. |
| L. 71-81: you should precise the tooth loci investigated in the previous publications | good point; for reference 25, we remain vague ('various' measurements) as in this paper, data from several other studies are collated and the statment refers to the total of these measeurements – if would become quite messy if we would list all species and the respective measurement locations | addition made l. 71: In hypselodont teeth (both incisors and cheek teeth)<br>addition made l. 74: show cheek tooth wear rates<br>addition made l. 78: enamel hardness (of unspecified location on the teeth)<br>addition made l. 82: Preliminary data from microhardness testing of rabbit enamel (measured on the chewing surface of both incisors and cheek teeth)<br>addition made l. 83: to various enamel hardness measurements |
| L. 177, 180, 186: write "Shapiro-Wilk" | good point, thank you | changes made l. 181, 184, 190: Shapiro-Wilk |
| L. 313: write "We thank…" | indeed, thank you | change made l. 333: We thank |

*****************************************************************************

## Reviewer 2:

Reviewer #2: This is a well-executed, concise experimental study on enamel abrasion in the evergrowing incisors of Myocastor and the non-evergrowing lower incisors of Bos taurus. The authors have performed enamel hardness measurement by indentation and a brushing experiment with three different abrasive agents plus a control group.
The methodology is well described, the results are clearly outlined and the discussion is supported by the results.

| Reviewer's comment (lines refer to the original submission) | Our response | Changes made in the text (lines refer to the revised version) |
|---|---|---|
| When discussing the different wear rates in the incisors of Myocastor and Bos, the authors might consider the difference in enamel thickness. Rodent incisors generally have a comparatively thin labial enamel cover which together with the underlying softer dentine provides a sharp cutting edge. Enamel microstructure of rodent incisor enamel is among the most complex, if not the most complex, schmelzmuster within mammals, and it is assumed that this is closely related to the high stresses that occur in the enamel during the gnawing process (in order to prevent the enamel from failure). So the thinness of the rodent enamel may play an important role in the higher abrasive rate. | We do not think that the thinness was decisive in this experiment (we stressed this point more in the methods), but the reviewer is right that in a live setting, the thinness of the enamel in occlusion in the incisors plays a role.<br>Yet, the same highh wear rates also apply to hypselodont cheek teeth e.g. in rabbits or guinea pigs. We made some additions to make sure the concern was covered in the manuscript. | addition made l. 178: In all other cases, the enamel layer submitted to testing was not worn off completely.<br>addition made l. 241: The hardness measurement and the wear challenge were applied to  enamel from the (labial) side of the tooth. In live animals, wear will occur at the occlusal surface of the tooth – in nutria, mainly due to contact with the other incisor, and in cattle (with no maxillary incisors) due to contact with food. Therefore, the wear measured our in vitro system is unlikely to resemble wear in live animals. Notably, the enamel microstructure, which is particularly complex in rodents [47], will serve to reinforce the enamel in the functional chewing direction and not necessarily against unnatural abrasion from the side. |
| Another factor is found in the antagonistic incisor – during the gnawing process, the incisor occlusal surfaces get in contact and therefore experience increased abrasion (self-sharpening mechanism). In the cattle, there are no upper incisors and therefore no tooth-tooth contact. Perhaps the authors can add a couple of sentences to the discussion considering these aspects. | good point, added | addition made l. 242: In live animals, wear will occur at the occlusal surface of the tooth – in nutria, mainly due to contact with the other incisor, and in cattle (with no maxillary incisors) due to contact with food. Therefore, the wear measured our in vitro system is unlikely to resemble wear in live animals. |
| If I got it right, the enamel samples from the nutria incisors were taken from the labial side, and were brushed from that side in the experiment. However, in the living animal enamel abrasion occurs on the occlusal surface of the incisors, which is at an angle to the labial side of the enamel cover. As the authors state in the introduction, enamel hardness is also thought to be dependent of the orientation of the enamel crystallites (e.g. radial enamel of the outer portion in rodent incisors is assumed to be particularly resistant against wear due to the steep inclination of the enamel prisms [wear occurs at a right angle to the c-axes]). When discussing the differences of the experimental enamel abrasion rate, this aspect would be worthwhile to be mentioned. | We included the fact of using the labial side in the methods. | addition made l. 130: samples from the labial side of the incisors were prepared<br>addition made l. 241: The hardness measurement and the wear challenge were applied to  enamel from the (labial) side of the tooth. In live animals, wear will occur at the occlusal surface of the tooth – in nutria, mainly due to contact with the other incisor, and in cattle (with no maxillary incisors) due to contact with food. Therefore, the wear measured our in vitro system is unlikely to resemble wear in live animals. Notably, the enamel microstructure, which is particularly complex in rodents [47], will serve to reinforce the enamel in the functional chewing direction and not necessarily against unnatural abrasion from the side. |
| Line 93: …(Myocastor coypus), with hypselodont (evergrowing) teeth. Replace "teeth" with "incisors", bescause the cheek teeth of Myocastor are not evergrowing. | good point | change made l. 95: with hypselodont (ever-growing) incisors |

******************************************************************************
